# The Importance of Facilitating Goal-Concordant Care (GCC) in a Pandemic: The MD Anderson Experience with hospitalized COVID-19 positive patients

**DOI:** 10.21203/rs.3.rs-2968661/v1

**Published:** 2023-06-12

**Authors:** Mayoora Muthu, Shalini Dalal, Marina George, Cesar Simbaqueba Clavijo, Caitlin Lenz, Nico Nortje

**Affiliations:** The University of Texas MD Anderson Cancer Center

**Keywords:** goals of care, goal concordant care, advance care planning, covid-19, coronavirus

## Abstract

**Purpose:**

Provider-patient communication (PPC) about goals of care (GOC) facilitates goal-concordant care (GCC) delivery. Hospital resource limitations imposed during the pandemic made it vital to deliver GCC to a patient cohort with COVID-19 and cancer. Our aim was to understand the population and adoption of GOC-PPC along with structured documentation in the form of an Advance Care Planning (ACP) note.

**Methods:**

A multidisciplinary GOC task force developed processes for ease of conducting GOC-PPC and implemented structured documentation. Data were obtained from multiple electronic medical record elements, with each source identified, data integrated and analyzed. We looked at PPC and ACP documentation pre and post implementation alongside demographics, length of stay (LOS), 30-day readmission rate and mortality.

**Results:**

494 unique patients were identified, 52% male, 63% Caucasian, 28% Hispanic, 16% African American and 3% Asian. Active cancer was identified in 81% patients, of which 64% were solid tumors and 36% hematologic malignancies. LOS was 9 days with a 30-day readmission rate of 15% and inpatient mortality of 14%. Inpatient ACP note documentation was significantly higher post-implementation as compared to pre-implementation (90% vs 8%, P < 0.05). We saw sustained ACP documentation throughout the pandemic suggesting effective processes.

**Conclusions:**

The implementation of institutional structured processes for GOC-PPC resulted in rapid sustainable adoption of ACP documentation for COVID-19 positive cancer patients. This was highly beneficial for this population during the pandemic, as it demonstrated the role of agile processes in care delivery models, which will be beneficial in the future when rapid implementation is needed.

## BACKGROUND

The Covid-19 pandemic has stressed healthcare systems across the globe, with hospitals facing resource challenges such as low bed capacity, ventilator shortages, staffing inadequacies and scarcity of personal protective equipment (PPE), to name a few. It has been found that there is futility in cardiopulmonary resuscitation in the Covid-19 patient population, with one retrospective multi-hospital study showing that patients with Covid-19 who suffered from in-hospital cardiac arrest had 100% mortality regardless of their baseline comorbidities, illness severity, and location of arrest, with 81% of these patients being on a mechanical ventilator prior to arrest and a majority of the cardiac arrests (84.1%) occurring in the ICU setting.^[1]^

While it has always been of paramount importance to prioritize timely goals of care (GOC) conversations, the gravity of the pandemic and the known futility of resuscitation in this setting, placed further urgency on timely delivery of goal concordant care for our unique population of patients with cancer plus Covid-Effective and empathetic communication about disease prognosis, patient values and preferences, and treatment options is vital in delivering goal-concordant care (GCC). While appropriate discussions about advance care planning (ACP) are best initiated in the outpatient setting by primary oncologists, an admission to the hospital presents an important opportunity to re-evaluate and continue GOC discussions, as it signals a change in the trajectory of the patient’s illness, giving increased relevance to these conversations. It is recorded that 99% of clinicians believe that GCC discussions are important^[2]^, however only 29% of clinicians report having such conversations.^[3]^ It is also worthwhile to note that only roughly 11% of patients report having GOC conversations with their providers^[4]^, though 92% of Americans indicated they would be comfortable having GOC and End-of-Life (EoL) discussions with their provider.^[3]^ Inconsistencies with care preferences has been associated with higher medical costs and lower quality of care for the patient.^[4, 5]^ Literature indicates that timely GOC contributes to better care experience by the patient,^[6, 7]^ longer survival,^[8]^ better quality of life,^[8, 9, 10]^ and fewer depressive symptoms by patients.^[8, 11]^ Now, more than ever, prioritizing timely GOC conversations and ensuring delivery of goal-concordant care is important, as we strive to respect the wishes of patients who do not prefer higher levels of care at EoL, while efficiently navigating potential shortages in resources and effectively steering resource allocation. Our primary aim is thus to give a global overview of our experience in delivering goal concordant care to the Covid-19 patient population within a cancer institution.

## PROCESS

At the direction of institutional leadership, a multidisciplinary GOC task force was created to accelerate the ongoing work of engaging patients with timely GOC conversations on March 17, 2020. This taskforce included medical oncologists, intensivists, ethicists, palliative care physicians, internal medicine hospitalists, nursing, case managers, and social workers. The task force convened daily to create appropriate criteria and workflow for the inpatient cancer population, to develop virtual training and allocating resources to support primary oncologists in initiating these sensitive yet essential conversations. Additionally, the task force was responsible for creation of standardized ACP note templates, to capture essential information related to goal-concordant care. A day later, March 18, 2020, a national emergency was announced due to the rapid spread of Covid-19. The institution set up a designated Covid-19 unit and our first Covid-positive patient was admitted on March 24, 2020. This unique turn of global events prompted the initiation of a separate work stream for GOC on the Covid-19 unit.

Following initial review, the Covid-19 GOC team assessed challenges in the current process, strategized and proposed an updated workflow to tailor delivery of GCC to our distinctive population of Covid-19 patients with cancer. This new workflow included daily multidisciplinary virtual rounds/discussions with team members including nursing, oncologists, hospitalists, ethicist, physical therapy/occupational therapy, social worker and case management. This multidisciplinary method was taken to ensure that a holistic approach was utilized in determining each patient’s clinical condition, performance status, and severity of cancer and Covid-19 illness, and urgency for GOC conversation. A workflow process included a 3-tiered model for GOC conversations in the Covid-19 unit (Table I), which included the new GOC-Rapid Response Team (RRT). The RRT included the attending physician, palliative care physician and an ethicist, with the ability to respond within thirty minutes, if needed.

On April 24, 2020 the GOC team for the Covid-19 unit was formalized. All patients admitted to the Covid-19 unit were required to have a GOC conversation documented at some point during hospital admission, with preference given to documentation within first 24 hours of admission to the Covid-19 unit. After the initial GOC conversation, any acute change in condition would appropriately necessitate a follow-up GOC conversation with either the patient or family members (medical Power of Attorney [mPOA]/surrogate/legal next of kin). We instituted this workflow during a pilot period from April 24, 2020 through May 24, 2020 and continued the efforts from May 25, 2020 onwards to present day, making efforts to measure sustainability of this care model through January 24, 2021 (Figure I).

This research was performed as part of the institutional Data-Driven Determinants for COVID-19 Oncology Discovery Effort (D3CODE), IRB-approved protocol 2020–0348. Data were obtained from structured and unstructured electronic medical record elements, clinical note text, and ACP note documentation. Each source was identified, data integrated and analyzed using the Palantir Foundry platform (Syntropy), part of the Context Engine Data Management System at the MD Anderson Cancer Center (MDACC). Additionally, for some areas of our research, which required manual data analysis, we utilized data that were collected and managed using REDCap electronic data capture tools hosted at MDACC.^[12, 13]^ REDCap (Research Electronic Data Capture) is a secure, web-based software platform designed to support data capture for research studies, providing 1) an intuitive interface for validated data capture; 2) audit trails for tracking data manipulation and export procedures; 3) automated export procedures for seamless data downloads to common statistical packages; and 4) procedures for data integration and interoperability with external sources.

## RESULTS

In our cancer institution, 494 unique patients who required hospitalization to the Covid-19 unit were identified from March 24, 2020 through January 24, 2021. 81% of patients admitted had an active cancer diagnosis, while the other 19% either had non-active cancer or cancer of indeterminate/unspecified origin. Of the 81% active cancers, 36% of patients had underlying active hematologic malignancies, and 64% had active solid tumor malignancies. 4.5% of total admitted patients were identified as having a cancer involving the respiratory tract.

Other high-risk comorbidities identified included hypertension (72%), chronic kidney disease/end-stage renal disease (45%), diabetes mellitus (44%), chronic obstructive pulmonary disease (17%), congestive heart failure (16%), asthma (13%), venous thromboembolism (12%) and obesity (12%). Mean patient age was 59, with median being 61. Gender distribution showed 52% of patients being male and 48% of patients being female. Race and ethnicity demographics showed 63% of patients identified as Caucasian, 28.6% as Hispanic/Latino, 16.2% as African American and 2.7% as Asian.

Inpatient average length of stay (LOS) was 9 days, and 30-day readmission rate was 15%. Inpatient Covid-19 mortality during this time was 14%. Of the patients that expired during their hospitalization for Covid-19 in this timeframe, 90.4% were Do Not Resuscitate (DNR), 82.2% opted for comfort care, and 9.6% remained full code status, expiring after a terminal code blue event (Figure II). Referral to social worker was 53.4%, supportive care service was 15.4%, to spiritual services it was 12.6%, and to psychiatry it was 0.6% (Figure III).

During the timeframe of our study, a mean of 90% of patient encounters had ACP note documentation, with 6 out of 11 of the study period months having greater than 90% ACP note documentation (Figure IV). We noted that this practice sustained even past our pilot period and through our peak census times. During the pre-implementation period (March 24, 2020 through April 23, 2020), only 8% of Covid-19 patient encounters had ACP note documentation. Comparatively, on non-Covid hospitalized patients within our institution, ACP note documentation was recorded to be a mean of 58% for the same post-implementation time-period (Figure V). We also found that there was a correlation between age of patient and provider ACP note documentation within the first 24 hours of hospitalization of the Covid Unit, with the highest ACP note documentation rate being in patients greater than or equal to 81 years of age (51.85%) and the lowest ACP note documentation rate being in patients less than or equal to 30 years of age (22.58%).(Figure VI)

## DISCUSSION

It is challenging to make conclusive statements regarding pre- and post-GOC algorithm implementation outcomes for the Covid-19 patients, given that pre-implementation patient cohort consisted of patients (n = 29) admitted from March 24, 2020 through April 23, 2020 and post-implementation cohort included patients admitted from April 24, 2020 through January 24, 2021 (n = 465). However, our experience showed that with implementation of a daily multidisciplinary goal-concordant approach on the Covid-19 unit, a significant proportion of physicians had routine GOC conversations with patients and/or caregivers and documented their outcomes in the format of a templated ACP note (90%), which identified goals of cancer care as well as goals of Covid-19 care specifically. Our benchmark goal for ACP note documentation during this study period was 70%. Our benchmark goal as well as achieved ACP documentation rate of 90% substantially exceeds the 11% of patients reported as having GOC conversations with their providers in literature.^[4]^ This is further highlighted by our analysis showing 90.4% of those patients (or caregivers of patients) who expired opted for DNR status leading up to EoL, along with 82.2% of those patients electing to go the comfort care route.

Additionally, we found through literature search ^[14–17]^ that our inpatient mortality rate of 14% was amongst the lowest published hospitalized Covid-19 patient mortality rate, during a time when Covid-19 vaccination was not yet widely available or robustly implemented. We were able to extract data on illness severity for our Covid-19 cancer patient population during the study time period and found that 67.4% of patients required some degree of supplemental oxygen support, while 19.8% of patients required higher levels of non-invasive oxygen support (i.e., high-flow nasal cannula, non-rebreather mask, or non-invasive positive pressure ventilation), and 8.3% of patients ultimately required mechanical ventilation (Figure VII). Additionally, 22% of these patients were noted to have worsening oxygen requirements within the first seventy-two hours of hospital admission (Figure VIII). Maintaining a low inpatient mortality rate in patients with such high illness severity furthermore emphasizes the vital significance of utilizing an adept multidisciplinary care team for complex patient populations.

These figures demonstrate that early initiation of conversations regarding goal concordant care between patients, caregivers and providers have significant impact on EoL outcomes. The more traditional model of care in cancer medicine previously has been dichotomous, with curative or disease-modifying treatment offered primarily and palliative options only being discussed later in disease course. Including a selected team of experts in having these discussions, not only lowers the burden of responsibility of the primary treating physician, but also increases the support system for the patient/family/caregiver. Within our Covid-19 patient cohort for the study period, we found that 53.4% of patients were referred to social work for either medical power of attorney identification, living will documentation, hospice education, out-of-hospital DNR documentation, or other social/financial issues. 15.4% of patients were referred to our supportive/palliative care consultants for either pain/symptom management, assistance with GOC, or psychologic services. 12.6% of patients were referred to spiritual services.

This type of multidisciplinary approach affords patients/family/caregivers the opportunity to look at their current situation from more than just the medical perspective. Palliative care specialists are skilled in EoL issues and questions, while ethicists are skilled in the methodology of facilitated conversations. Ethicists also ensure that different value systems are respected and integrated into the conversation. Thus, integrating these specialists into GOC conversations, along with the primary inpatient teams and oncologists, provides greater value for patients/caregivers, whose decision-making is optimized when they are presented with a global view of their treatment options and overall prognosis.

Our model suggests more compassionate outcomes when utilizing a goal-concordant approach to those patients with cancer plus multiple comorbidities including Covid-19, so that they are educated early in the disease process on the option of a palliative approach and thus, may receive timely and high-quality palliative care when appropriate. Accordingly, we conclude that there is notable utility in implementing a multidisciplinary approach to goal concordant care in the hospitalized cancer population with Covid-19 illness. This concept likely has broader benefit in fundamental application to all hospitalized cancer patients. Covid-19 will likely continue its significant impact on our vulnerable immunocompromised community of patients, thus as clinicians, it is our ethical responsibility to provide patients and caregivers with the tools and education to make informed decisions regarding end-of-life care.

## Figures and Tables

**Figure 1:**
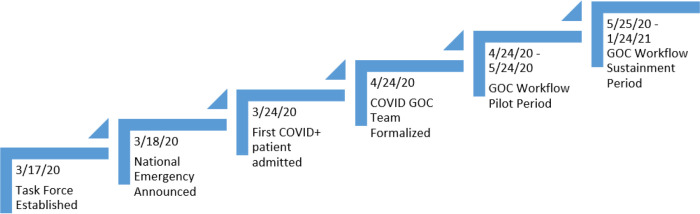
Timeline of GOC Implementation

**Figure 2:**
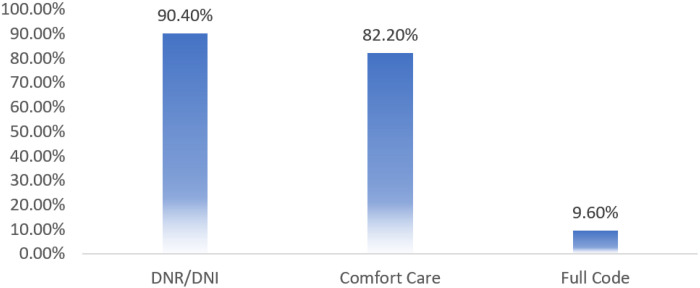
Code Status in the Deceased Cohort of Hospitalized Covid Patients in a Cancer Institution

**Figure 3:**
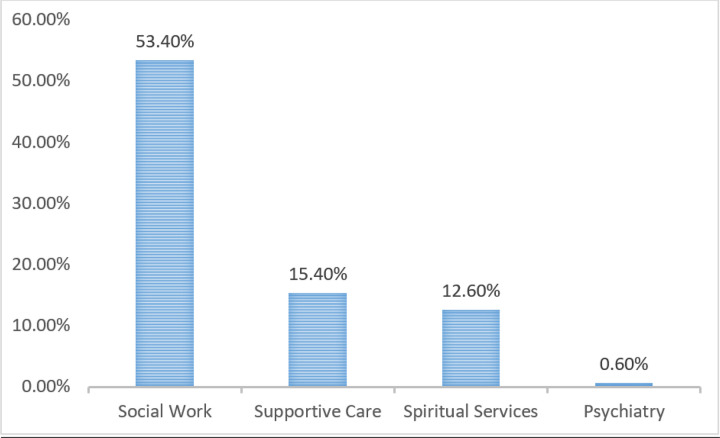
Percentage of Covid-19 Patients Referred to Supportive Services in a Cancer Institution

**Figure 4:**
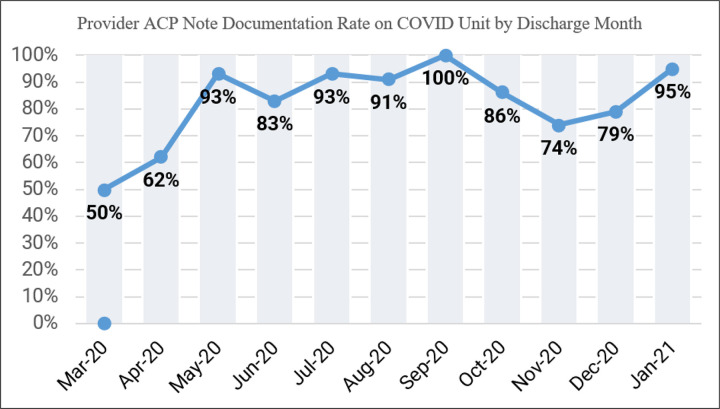
Provider ACP Note Documentation Rate on the COVID Unit in a Cancer Institution

**Figure 5:**
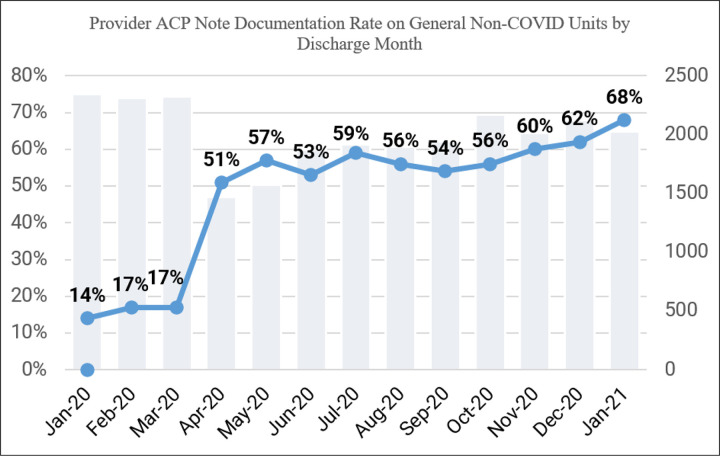
Provider ACP Note Documentation Rate on the General Inpatient Units in a Cancer Institution

**Figure 6:**
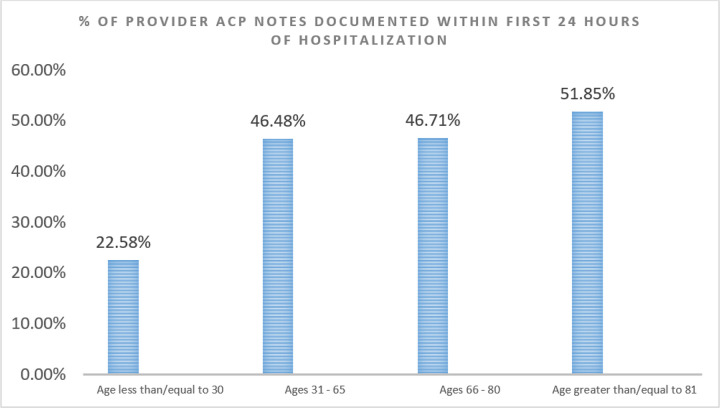
Provider ACP Note Documentation Rate (by Age Group) on the Covid Unit in a Cancer Institution

**Figure 7:**
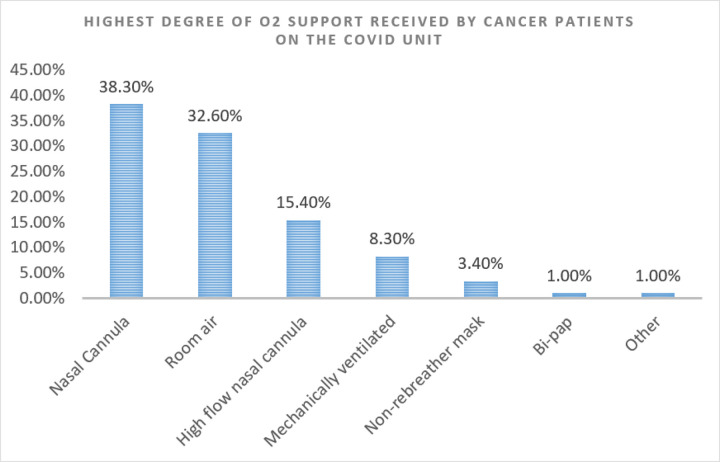
Illness severity (by O2 status) on the Covid Unit in a Cancer Institution

**Figure 8:**
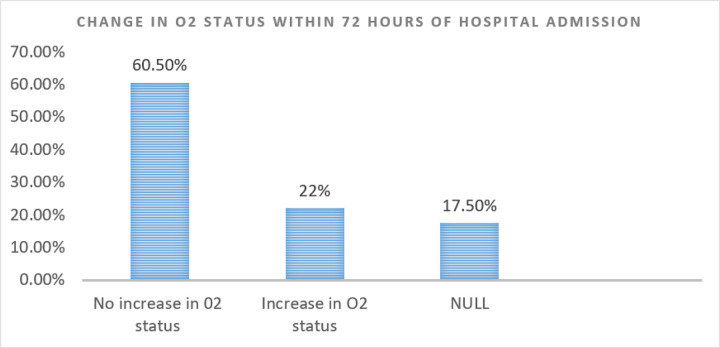
Illness Severity by Deterioration Index on the Covid Unit in a Cancer Institution *NULL = if length of stay is less than 72 hours or if O2 status was not consistently recorded within 72 hours

**Table I:** 3-Tiered GOC Model

MODELS OF GOC conversations	Primary Oncologist led GOC (Same day)	Co-managed GOC discussion (1–2 days)	GQC-RRT (Rapid Response Team) (Urgent/Same day/24 hours)
Why	To establish clear GOC for patient	Oncologist with peer support; complicated medical situation or family dynamics	Clear GOC absent, patient is declining
Patient population	All pts admitted within past 24 hours with risk of escalation level 2 or 3	Any patient with complex clinical or psychosocial needs	Any patient needing timely, integrated approach [Primary oncologist supported]
who is present	Primary oncologist/On-call oncologist	Primary oncologist/On-call Oncologist Palliative care, Social work	Primary Oncologist/On-call oncologist GOC RRT (Social work. Ethics, Palliative tare)
Aim	To give clear information and clarity the patient’s wishes to ensure goal-concordant care, Requires periodic re-evaluation,	To give clear information and clarify the patient’s wishs in the context of the complex ongoing issues. Requires periodic ne-evaluation.	To give rapid, coordinated, clear information and clarify the patient’s wishes, anticipating imminent define. Requires periodic re-evaluation.
Conversation model	Self-directed Faculty education conversation guide available	Performed hy primary attending with Palliative care and/or social work using the briefing/debriefing model	Performed by primary attending with Palliative care, ethics, and social work using the briefing/debriefing model
How to obtain	Self directed	Consult to palliative care via **EPIC**	Reach out to the on-call Case Management
Documentation	Primary oncologist documents available in the ALP tab	Primary Oncologist or Palliative care documents in the ACP tab	Primary Oncologist or Palliative care documents in the ACP tab
Support	Inpatient medical director	Inpatient medical director Palliative care leader	Case Management Palliative care leader Clinical Ethics
